# Leucoma salicis nucleopolyhedrovirus (LesaNPV) genome sequence shed new light on the origin of the *Alphabaculovirus orpseudotsugatae* species

**DOI:** 10.1007/s11262-024-02062-x

**Published:** 2024-04-09

**Authors:** Martyna Krejmer-Rabalska, Lukasz Rabalski, Maciej Kosinski, Iwona Skrzecz, Jadwiga Ziemnicka, Boguslaw Szewczyk

**Affiliations:** 1 Laboratory of Recombinant Vaccines, Intercollegiate Faculty of Biotechnology, University of Gdansk and Medical University of Gdansk, 80-307 Gdansk, Poland; 2 Biological Threats Identification and Countermeasure Center, General Karol Kaczkowski Military Institute of Hygiene and Epidemiology, 24-100 Pulawy, Poland; 3 Department of Forest Protection, Forest Research Institute, 05-090 Sekocin Stary, Poland; 4 Department of Biological Control and Quarantine, Institute of Plant Protection, 60-318 Poznan, Poland

**Keywords:** *Leucoma salicis*, LesaNPV, Alphabaculovirus, DapuNPV, OpMNPV, Common ancestor

## Abstract

LesaNPV (Leucoma salicis nucleopolyhedrovirus) is an alphabaculovirus group Ib. Potentially, it can be an eco-friendly agent to control the white satin moth *Leucoma salicis* population. In this study, we have established the relationship between LesaNPV and other closely related alphabaculoviruses. Environmental samples of late instar of white satin moth collected in Poland infected with baculovirus have been homogenized, polyhedra were purified and subjected to scanning and transmission electron microscopy. Viral DNA was sequenced using the Illumina platform and the whole-genome sequence was established by de novo assembly of paired reads. Genome annotation and phylogenetic analyses were performed with the use of bioinformatics tools. The genome of LesaNPV is 132 549 bp long with 154 ORFs and 54.9% GC content. Whole-genome sequencing revealed deletion of dUTPase as well as ribonucleoside reductases small and large subunits region in LesaNPV genome compared to Dasychira pudibunda nucleopolyhedrovirus (DapuNPV) and Orgyia pseudotsugata multiple nucleopolyhedrovirus (OpMNPV) where this region is complete. Phylogenetic analysis of *Baculoviridae* family members showed that LesaNPV is less divergent from a common ancestor than closely related species DapuNPV and OpMNPV. This is interesting because their hosts do not occur in the same area. The baculoviruses described in this manuscript are probably isolates of one species and could be assigned to recently denominated species *Alphabaculovirus orpseudotsugatae,* historically originating from OpMNPV. This finding could have significant implications for the classification and understanding of the phylogeographical spread of baculoviruses.

**Supplementary Information:**

The online version contains supplementary material available at 10.1007/s11262-024-02062-x.

## Introduction

Recently, the new classification of arthropod-specific large dsDNA viruses (nuclear arthropod large DNA viruses, NALDVs) has been introduced based on the presence of homologs of genes coding for conserved proteins involved in the mechanism of baculovirus primary infection (*per os* infectivity factors, *pif*s) and other shared features. The class *Naldaviricestes* contains *Baculoviridae, Nudiviridae, Hytrosaviridae*, and *Nimaviridae*. The former three—*Baculoviridae, Nudiviridae, Hytrosaviridae—*are higher arranged into *Lefavirales* order according to the late expression factors genes responsible for late-phase baculovirus infection present in the genomes of its members (ICTV online meeting, October 2020 [1]).

The *Baculoviridae* family comprises large, rod-shaped viruses with circular, double-stranded DNA as a genetic material [2, 3]. They infect larval stages of insects, mainly butterflies (Lepidoptera), sawflies (Hymenoptera) and true flies (Diptera). Baculovirus genome ranges in size between 80 and 180 kbp and encodes 90–180 genes, 38 of which are so called “core genes”—present in every species of the *Baculoviridae* family [4, 5]. The classification of the *Baculoviridae* family consists of four genera due to genomic sequences and host-dependent evolution: *Alpha*-, *Beta*-, *Delta*- and *Gammabaculovirus* [6, 7]. During the life cycle, two genetically identical but phenotypically different forms of virions occur—occlusion-derived virions (ODVs) and budded virions (BVs). The first is responsible for insect-to-insect transmission, while the second for systemic infection within a host. ODVs are embedded in two types of occlusion bodies (OBs)—polyhedral-shape made of polyhedrin in nucleopolyhedroviruses (NPVs) and granular-shape built of granulin in granuloviruses (GVs) [3, 8]. Major structural occlusion body protein is highly conserved and enables baculoviruses to survive decades in the environment [9]. Alphabaculoviruses are lepidopteran-specific NPVs. Recently, baculovirus species nomenclature has been systematized. In brief, using the genus name followed by a single epithet describing the baculovirus host name from which it was originally isolated, e.g., Orgyia pseudotsugata multiple nucleopolyhedrovirus (OpMNPV) belongs to the *Alphabaculovirus orpseudotsugatae* species [10].

One of baculovirus characteristic features is a very narrow host range. Baculoviruses infect one or few closely related insect species and coevolved with their hosts [6]. Due to this unique specificity, most baculoviruses are considered as excellent candidates for biological pest control. From the environmental point of view, baculoviruses are safe alternatives for chemical pest control as their host range is generally restricted to one insect species or a few closely related species, not affecting other organisms like humans and beneficial animals, e.g., bees [11].

**Fig. 1:**
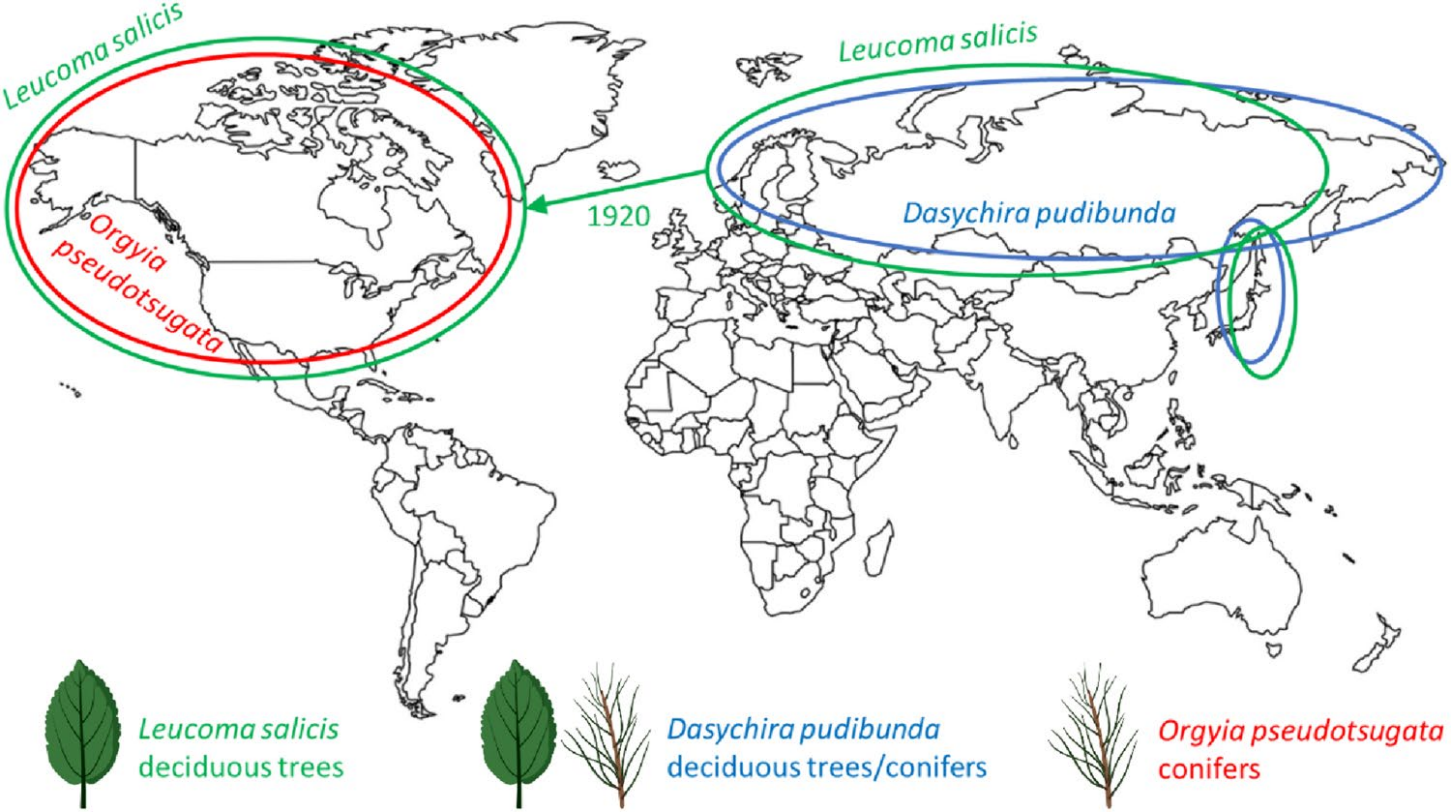
World distribution of insect hosts *Leucoma salicis*, *Dasychira pudibunda*, and *Orgyia pseudotsugata* of baculoviruses LesaNPV, DapuNPV, and OpMNPV with schematic representation of their food preferences (conifers or deciduous trees). *L. salicis* has been reported to have been introduced to North America in the 1920s (created in biorender.com)

The white satin moth *Leucoma salicis* L. (Lepidoptera, Erebidae, Lymantriinae) is a significant defoliator occurring mainly in Europe and Asia, but it was also introduced to North America in the 1920s [12–15] (Fig. 1). The caterpillars feed principally on the leaves of poplar (*Populus* spp*.*), including eastern cottonwood (*Populus deltoides* Bartram and Marshall) aspen as well as willow trees (*Salix* spp.) and less commonly on oak (*Quercus* spp.). They attack healthy trees, which makes them the primary pest and can lead to massive defoliations and weakened trees. One of the main natural enemies of this insect is baculovirus Leucoma salicis nucleopolyhedrovirus (LesaNPV) [16, 17]. For population control of white satin moth biopesticide based on LesaNPV can be used successfully [18]. According to short nucleotide sequences of three genes (*polh*, *lef8*, *pif-2*) available in the NCBI database, LesaNPV was annotated as *Alphabaculovirus* group I [16]. In the past, LesaNPV genetic characteristic has been analyzed [16], but whole genome and phylogeny have not been established. In our studies, we have described genomic sequence of LesaNPV, which we found highly similar to genomes of other alphabaculoviruses isolated from closely related to *Leucoma salicis* hosts—Orgyia pseudotsugata multiple nucleopolyhedrovirus (OpMNPV [19]) and Dasychira pudibunda nucleopolyhedrovirus (DapuNPV-ML1, Polish strain, [20]). Here, we characterize the relationship within the group of these three baculoviruses, wanted to extend the knowledge about them and discuss species affiliation of members of the *Baculoviridae* family based on host occurrence area and genetic sequences. We have also analyzed the sequence of host range factor-1 (hrf-1) present in LesaNPV genome.

## Materials and methods

### Virus purification and DNA extraction

Historical environmental samples (late instar of white satin moth *Leucoma salicis* collected in 1988 in Poland, near Poznań) were macerated with glass homogenizer in PBS buffer, until all parts beside hair and chitin fragments were in paste-like suspension. Homogenate was filtered through cheesecloth placed in 50-ml tubes. Samples were washed with PBS multiple times until only solid parts remained on the filter. PBS was added to the samples for a total volume of 45 ml. Samples were centrifuged 5000×*g* for 10 min, supernatant was discarded, and pellet was resuspended by vortexing in 40 ml of ddH_2_O. Samples were centrifuged in the same conditions and the supernatant was discarded. The pellet was resuspended in 40 ml of 0.5-M NaCl. Centrifugation was then repeated under identical conditions, the supernatant was again discarded, and the pellet was suspended in 2 ml of ddH_2_O.

The purified material was ultra-centrifuged on sucrose density gradient (40–65% w/w) 110000×*g* for 3 h at 4 °C. The band containing OBs was collected, diluted for final sucrose concentration of under 5%, and ultra-centrifuged again at 110000×*g* for 1 h. Pellets containing polyhedra were suspended in 200-µl ddH_2_O. Their presence was confirmed with light microscopy.

Isolated OBs were dissolved using Na_2_CO_3_ in a final concentration of 0.1 M (pH 10.0) for 30 min at 37 °C. After visual confirmation of sample transparency, they were neutralized with equal volume of Tris (pH 6.4). Viral DNA extraction was performed using MagAttract HMW DNA Kit (Qiagen) according to the manufacturer’s protocol.

### Ultrastructural analyses

For Transmission Electron Microscopy (TEM), the purified OBs were fixed twice, first in a 2% mixture of glutaraldehyde and cacodylate buffer for 15 min, and then in a 2% osmium tetroxide solution for 1.5 h. The fixed polyhedra were successively dehydrated in ethanol, acetone, and propylene oxide and then embedded in Epon 812 [21]. The Epon blocks were cut on an ultramicrotome and the obtained sections were contrasted with phosphotungstic acid and viewed in a JEM 1200 EXII transmission electron microscope. Using photographs at magnifications of 20,000–40,000 × showing cross-sections through polyhedra, measurements were made of the diameter of 100 polyhedra from each isolate. The size (diameter and length) and number of nucleocapsids on these sections were determined.

For Scanning Electron Microscopy (SEM), purified OBs were treated with acetone and incubated for 1 h at 25 °C. Then, sample was loaded in a metallic stub, dried overnight at 37 °C, coated with gold in a SputterCoater (Balzers) for 3 min, and then observed in a SEM Jeol 7001TTLS.

### Virus DNA sequencing and bioinformatic analyses

The DNA isolated from baculovirus found in satin moth *L. salicis* larvae was sequenced at the Medical University of Gdansk in Poland using MiSeq (Illumina). For genomic library preparation Nextera XT DNA sample prep kit (Illumina) was used.

In the sequencing process, we generated 680,438 paired-end reads, each with a length of 2 × 300 bases. The raw sequencing data were processed using Geneious Prime (Biomatters, http://www.geneious.com/), which included a de novo assembly of the reads. The trimming of these reads was conducted using the default settings within Geneious Prime, aimed at optimizing the balance between data quality and the retention of informative bases. Post-trimming, the sequencing data achieved an average Phred quality score (Q) greater than 30 for 93% of the bases. This metric indicates a base-call accuracy of at least 99.9% for the vast majority of the sequenced bases. The coverage depth across the assembled genome ranged from 520 to 734X, ensuring a robust representation of the target sequence in our analysis. On contigs that were created ORFs were identified by Glimmer3 (gene model pre-computed on OpMNPV genome) [22] and GeneMarkS (parameter: Intron less eukaryotic virus) [23] and tcode EMBOSS 6.5.7 [24]. The ORFs annotation with homologous protein search was performed using HMMER web server (phmmer, NR large collection, scoring matric BLOSUM62 and BLOSUM45) [25].

The phylogenetic analysis of the *Baculoviridae* family was performed on 38 core genes extracted from 96 baculovirus genomes available in the GenBank database. These genes were translated to amino acid sequences, separately aligned (Multiple Alignment using Fast Fourier Transform (MAFFT)), and concatenated to create a comprehensive dataset. The phylogenetic reconstruction was conducted using the IQ-TREE multicore version 2.2.2.6 COVID edition, a fast and effective maximum likelihood phylogeny software [26]. The chosen model for the analysis was Q.insect + F + I + G4, which was determined to be suitable for the dataset. The robustness of the resulting tree was assessed by performing 1000 ultrafast bootstrap replicates (UFBoot) [27].

The best scoring maximum likelihood tree was constructed using the specified model with CuniNPV as the outgroup to root the tree. Branch lengths were optimized, and the log likelihood of the consensus tree was calculated. The final tree presented in this study shows the percentage of bootstrap replicates supporting each clade next to the branches.

The evolutionary history of HRF-1 was inferred using the Maximum Likelihood method and JTT matrix-based model [28]. The tree with the highest log likelihood (− 926.49) is shown. The percentage of trees in which the associated taxa clustered together is shown next to the branches. Initial tree(s) for the heuristic search were obtained automatically by applying Neighbor-Join and BioNJ algorithms to a matrix of pairwise distances estimated using the JTT model and then selecting the topology with superior log likelihood value. The rate variation model allowed for some sites to be evolutionarily invariable ([+ *I*], 12.23% sites). This analysis involved 15 amino acid sequences. All positions with less than 95% site coverage were eliminated, i.e., fewer than 5% alignment gaps, missing data, and ambiguous bases were allowed at any position (partial deletion option). There were a total of 73 positions in the final dataset. Evolutionary analyses were conducted in MEGA X [29].

Bioinformatic analyses, such as nucleotide or amino acid alignments (MAFFT), were performed using Geneious Prime (Biomatters Inc., New Zealand).

### Nucleotide sequence distance calculation

Kimura two-parameter (K2P) distances were calculated for the 38 core genes. Nucleotide sequences of 38 core genes were extracted from LesaNPV, DapuNPV, OpMNPV, HycuNPV, and DijuNPV, separately aligned (Multiple Alignment using Fast Fourier Transform (MAFFT) in Geneious Prime) and concatenated to create a comprehensive dataset. Gamma shape parameter (alpha) was calculated in MEGA X and then included in the calculation of K2P nucleotide distance values in MEGA X. Gaps within the alignment was treated as pairwise deletions.

## Results

### Electron microscopy studies

**Fig. 2:**
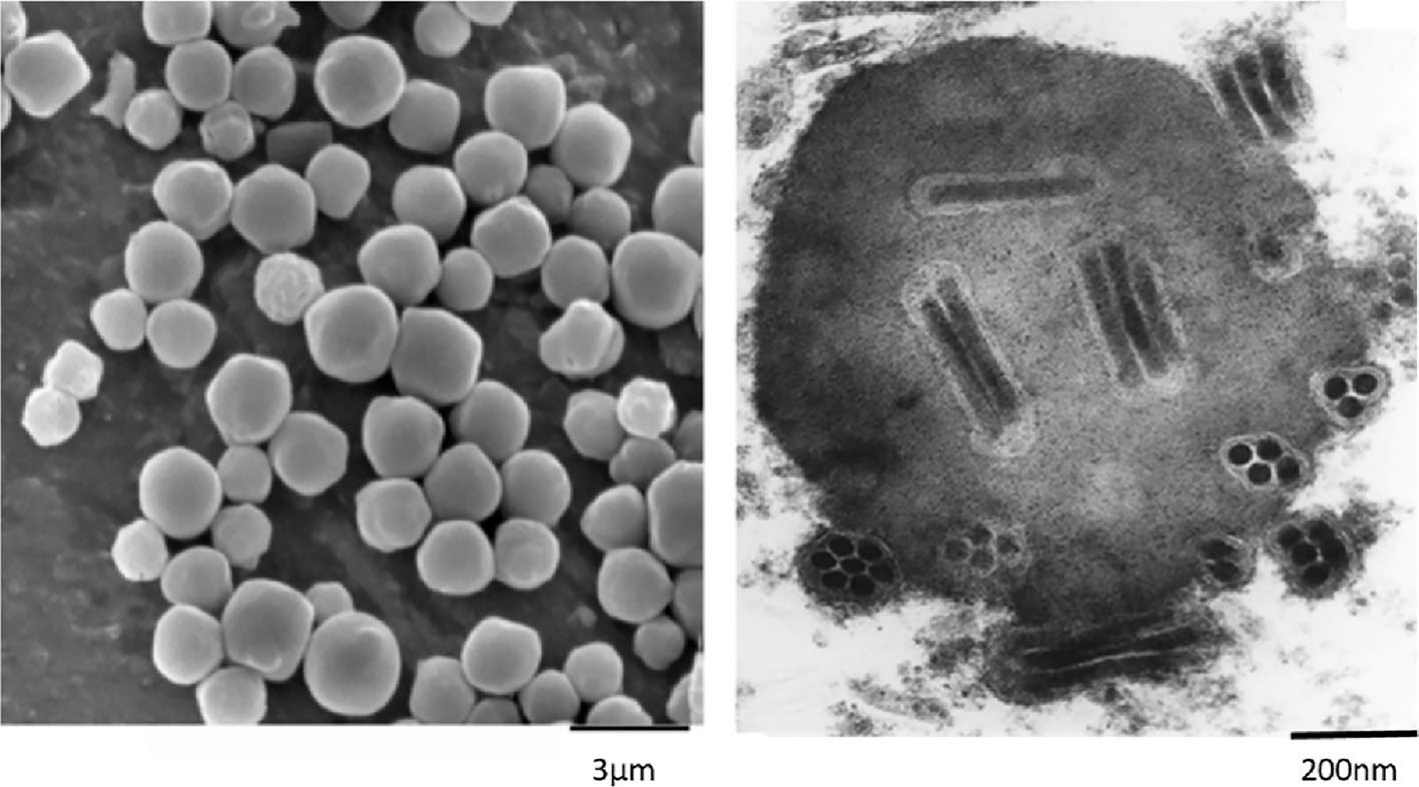
Scanning (SEM, left panel) and Transmission Electron Microscopy (TEM, right panel) photographs of LesaNPV. Multiple nucleocapsids are embedded in polyhedral occlusion bodies

Electron Microscopy analysis of LesaNPV morphology indicated that the shape of OBs is typical for nucleopolyhedroviruses (Fig. 2). Scanning Electron Microscopy (SEM) revealed that OBs have irregular shapes and a diameter around 1.4–3 µm. The cross-sections visualized in Transmission Electron Microscopy (TEM) showed that polyhedra have around 2–15 virions packaged inside with multiple (2–7) nucleocapsids embedded within the envelope of the virion. The size of one nucleocapsid is approximately 23–40 nm in diameter and 230–290 nm long.

### General characteristics of LesaNPV genome

**Fig. 3:**
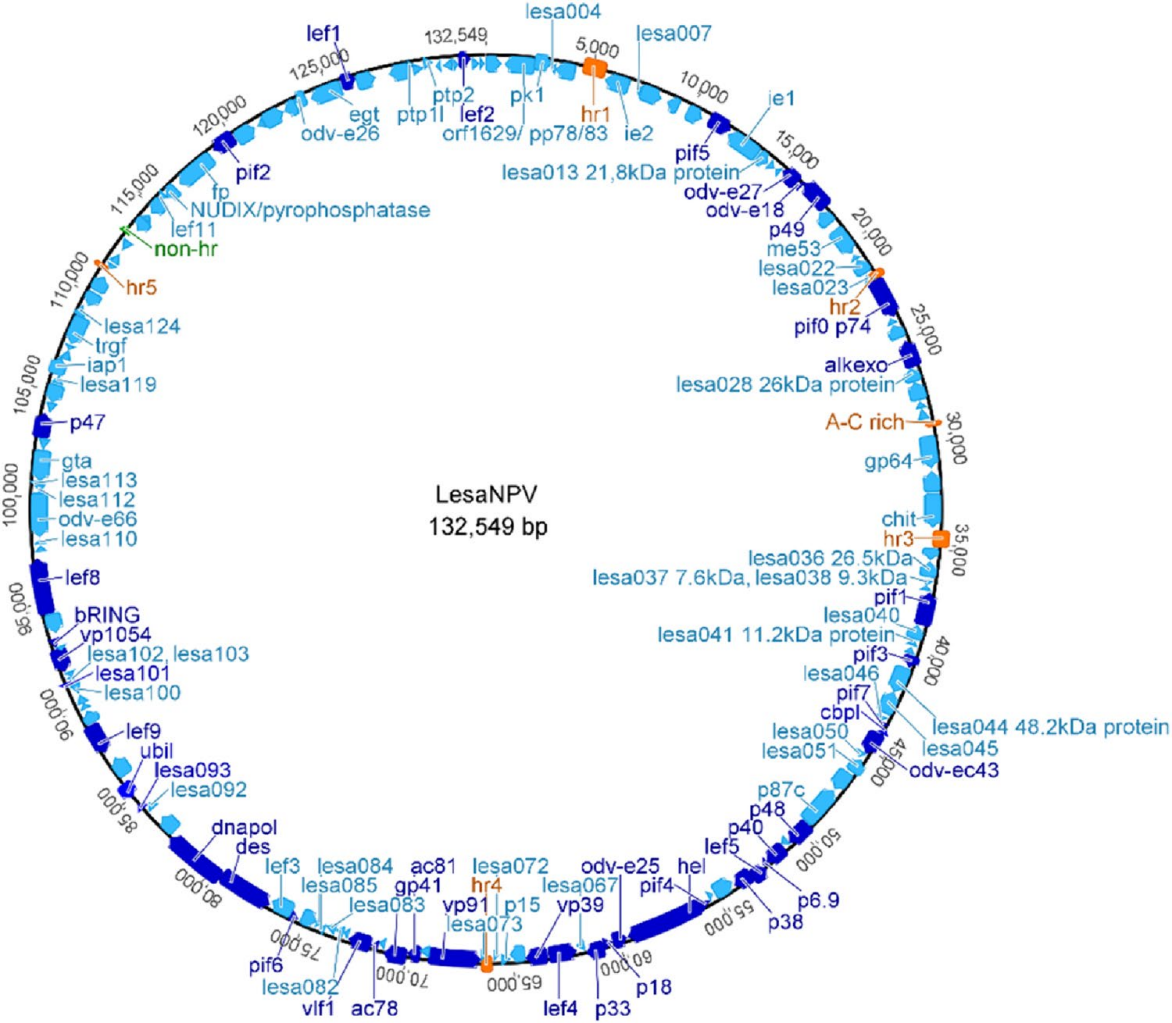
Circular map of LesaNPV genome. Arrows indicate the orientation of putative ORFs, marked in light blue. Core genes are marked in dark blue, *hrs* in orange, and *non-hr* region in green

The complete circular LesaNPV (characterized in this study is available under GeneBank accession number: OR759961) genome is 132 549 bp long (Fig. 3) and possesses 54.9% GC pairs, which is similar to DapuNPV-ML1 (GenBank accession no: KP747440)—54.4% and OpMNPV (GenBank accession no: NC_001875) 55.1%. The number of putative open reading frames (ORFs) is equal to 154, which is 7 less than DapuNPV-ML1 and 2 more than OpMNPV. The analysis and comparison of gene content of all three genomes are presented in Table S1. Among all the genes, 33 encode structural proteins, 9 *per os* infectivity factors, 14 are responsible for transcription, and 14 for replication, 31 genes encode auxiliary proteins and 53 ORFs have unknown functions. The ratio of identified *orf*s in forward orientation vs. reverse orientation is 71:83. All 38 baculovirus core genes have been identified in LesaNPV genome (marked in dark blue in Fig. 3). There are ORFs of unknown function that do not have homologs in other baculoviruses, not only DapuNPV or OpMNPV: *lesa092 and lesa093.* Five baculovirus repeated ORFs (*bro*) were found—*bro a*—*bro e,* while in DapuNPV there are three and one in OpMNPV*.* Differences in numbers, locations, and content of baculovirus *bro* genes between closely related species are their well-known characteristic [30]. Homologous repeats (*hrs*) have been found in almost all genomes of *Baculoviridae* family members. They are built of direct repeats, imperfect palindromes, high AT content, and are similar to each other within one baculovirus genome. LesaNPV genome has five homologous repeats (*hrs1-hrs5*) and one *non-hrs* region (marked in orange and green, respectively, in Fig. 3), the same as DapuNPV and OpMNPV. The AC-rich region is between *gp64* and *p24* genes in all three genomes. It is composed of multiple repetitions of motif CAACAACACA and has a length of around 220 base pairs, whereas this intergenic region is the longest in OpMNPV (the schematic nucleotide alignment and GC/AT content of the intergenic region is presented in Fig. S1). The LesaNPV genome possesses conserved non-protein coding element (CNE) 156p long in position 7214–7370 bp characteristic for all alphabaculoviruses and responsible for baculovirus replication in transfected insect cells [31].

**Fig. 4:**
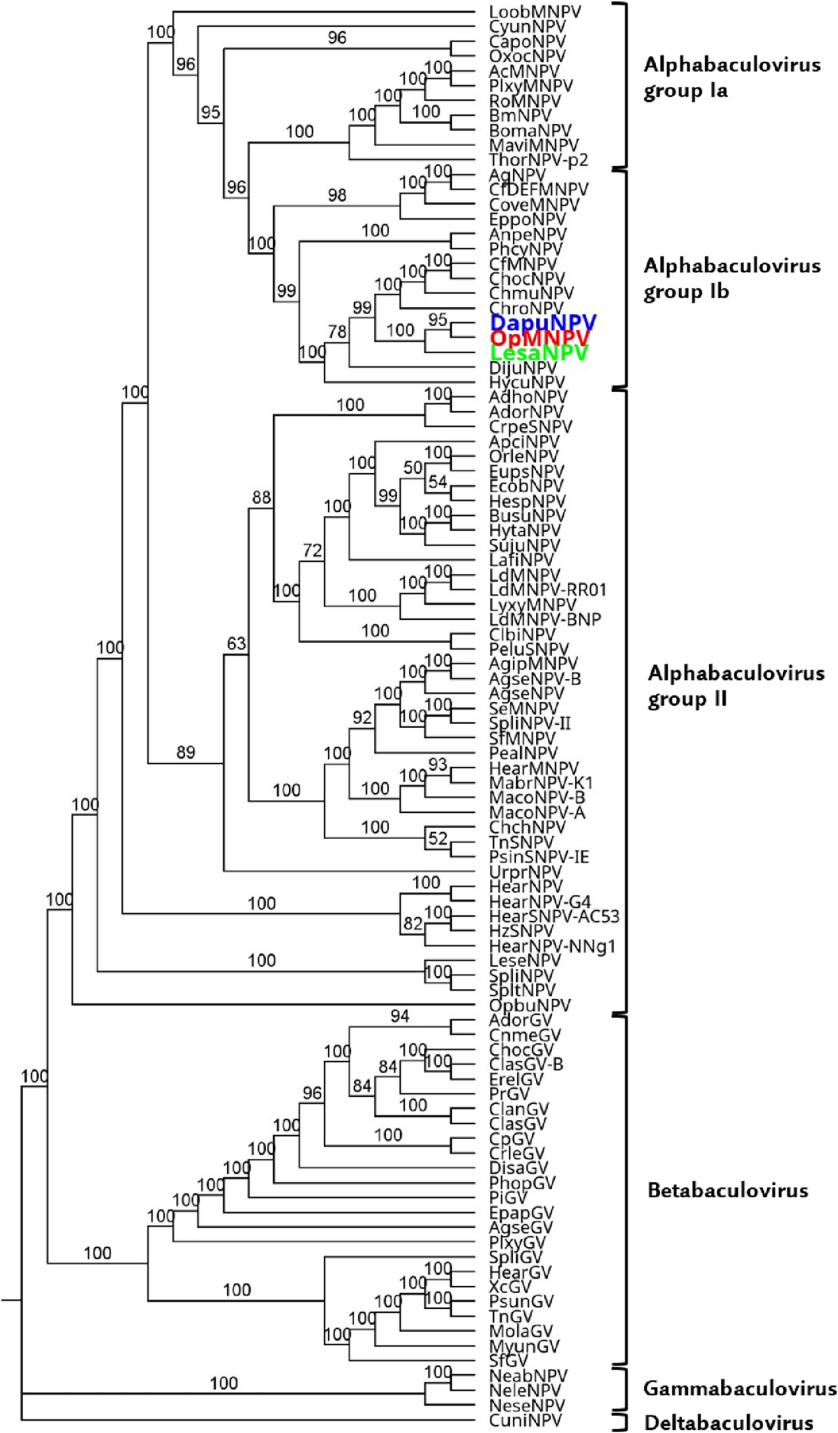
Phylogenetic cladogram illustrating the relationship within the *Baculoviridae* family. Maximum likelihood molecular analysis of 96 baculoviruses was performed based on concatenated sequences of 38 proteins encoded by core genes, utilizing IQ-TREE software with CuniNPV as the outgroup. The percentage of 1000 ultrafast bootstrap replicates supporting each clustered grouping of baculoviruses are indicated adjacent to the branches. The genera *Alphabaculovirus, Betabaculovirus, Gammabaculovirus*, and *Deltabaculovirus* are presented. A notable cluster includes LesaNPV (green), OpMNPV (red), and DapuNPV (blue), which demonstrate close evolutionary relationships

The presence of *gp64* and *F-protein* genes in the genome of LesaNPV allows for its classification as a representative of the *Alphabaculovirus* genus (group Ib). In addition, phylogenetic Maximum likelihood tree based on 38 core genes of other members of the *Baculoviridae* family presented in Fig. 4 indicates, according to new nomenclature, that it should be named as an isolate of *Alphabaculovirus orpseudotsugatae* species, because it clusters together with OpMNPV and DapuNPV. It appears that LesaNPV is more closely related to the common ancestor than the other two analyzed baculoviruses.

**Fig. 5:**
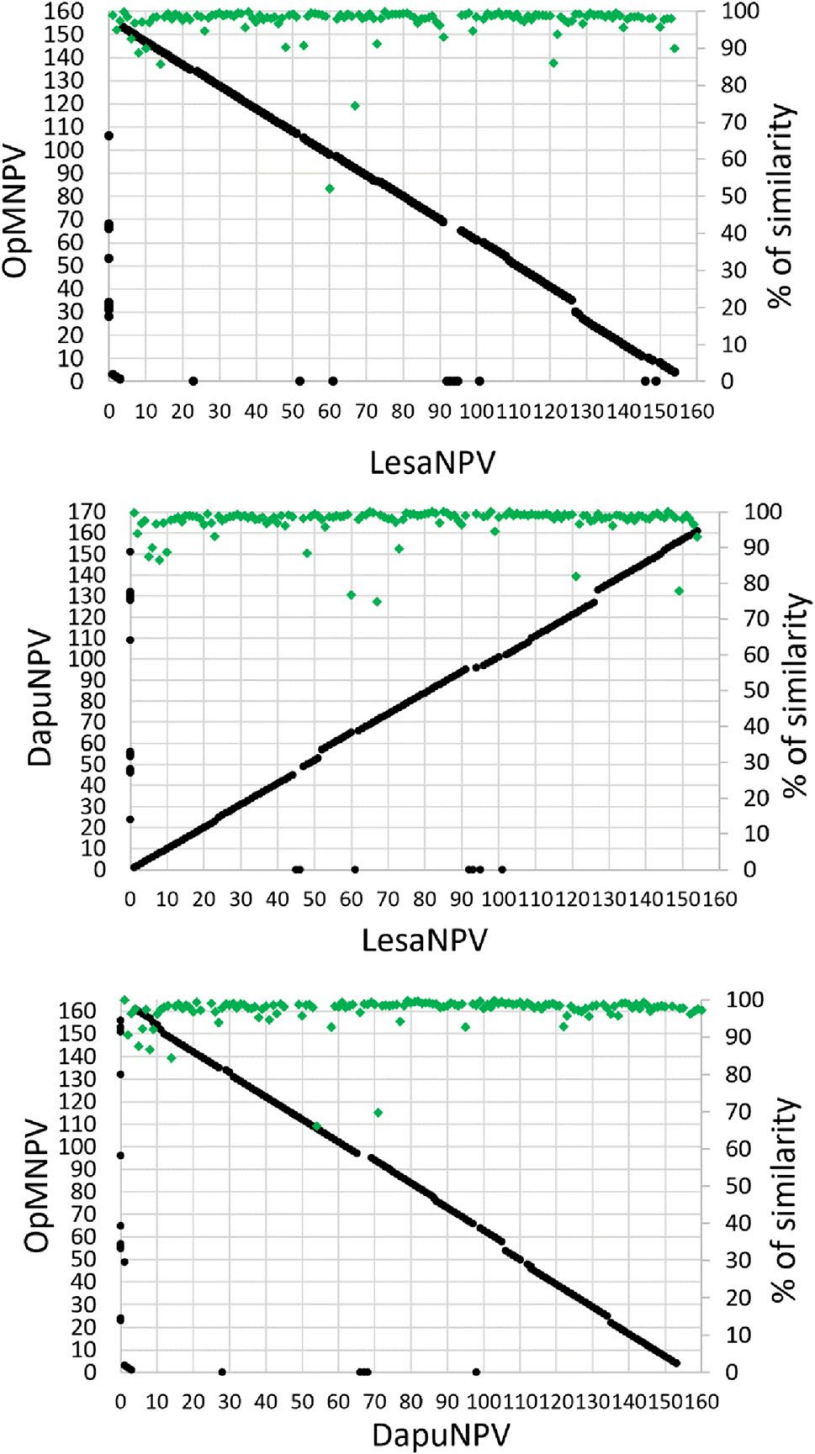
Gene-parity plot analysis. Gene-parity plots among LesaNPV, DapuNPV, and OpMNPV—three closely related alphabaculoviruses—where *polh* gene is considered a first gene. OpMNPV was adjusted by reverse complement of the whole genome so that *polh* is in the forward orientation, but original numbering of the ORFs stays unchanged. When there is no homolog ORF in the compared genome the ORF was treated as “0,” so is presented on “x”- or “y”-axis. Green dots indicate percentage of similarity of nucleotide sequences of homolog ORFs

Gene-parity plots of three closely related alphabaculoviruses are presented in Fig. 5. When there is no homolog ORF in the compared genome the ORF was treated as “0,” so is presented on “x”- or “y”-axis. All LesaNPV in comparison of OpMNPV, DapuNPV, and AcMNPV (Autographa californica multinucleopolyhedrovirus) ORFs are listed in Table S1 in supplementary material. Figure 5 shows high degree of similarity and collinearity in gene organization of LesaNPV, OpMNPV, and DapuNPV, as well as high percentage of similarity between nucleotide sequences of most of the genes in the analyzed genomes. No rearrangements can be observed in the genome structure.

Nucleotide sequence distances based on the Kimura two-parameter (K2P) of 38 core genes calculated for DapuNPV, LesaNPV, OpMNPV, and closely phylogenetically related Alphabaculovirus Ib baculoviruses Hyphantria cunea nucleopolyhedrovirus (HycuNPV) and Dione juno nucleopolyhedrovirus (DijuNPV) are presented in Table 1. Cut-off values for demarcation criteria based on partial *polh/lef8/lef9* for isolates from different species are 0.050 [8]. Values adjusted for all core genes set [32] that indicate the affiliation to defined species are below 0.021. All the K2P 38 core genes values for LesaNPV, OpMNPV, and DapuNPV distances among each other are below 0.021 (marked green in Table 1). For DijuNPV and HycuNPV, the nucleotide distances among five analyzed baculoviruses is much higher than cut-off value for one species for K2P 38 core gene. All the calculated values clearly indicate that LesaNPV, DapuNPV, and OpMNPV are members of one species, recently called *Alphabaculovirus orpseudotsugatae* and that HycuNPV and DijuNPV represent different species.

**Table 1:** Kimura two-parameter (K2P) pairwise distances calculated for the nucleotide sequences of the 38 core gene data

	DapuNPV	LesaNPV	OpMNPV	DijuNPV	HycuNPV
DapuNPV	–	**0,0121**	**0,0172**	0,2263	0,2496
LesaNPV	**0,0121**	–	**0,0162**	0,2268	0,2494
OpMNPV	**0,0172**	**0,0162**	–	0,2291	0,2578
DijuNPV	0,2263	0,2268	0,2291	–	0,2651
HycuNPV	0,2496	0,2494	0,2578	0,2651	–

Significant values given in bold

**Fig. 6:**
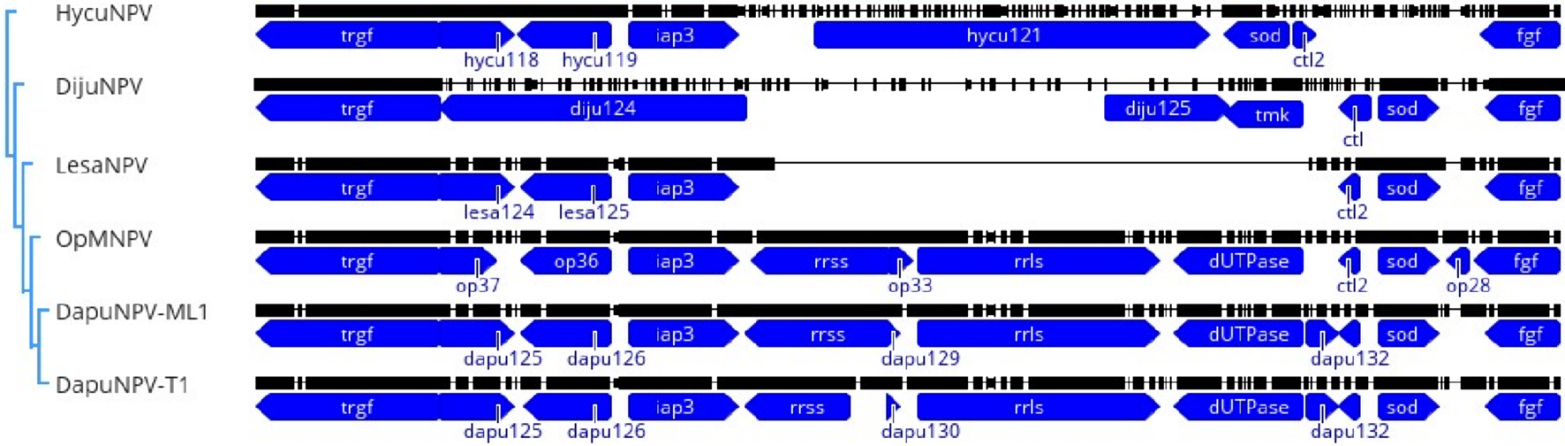
Schematic representation of an alignment of six nucleotide sequences of the region *trgf-fgf*, where *rrss-dUTPase* deletion occurs in LesaNPV in contrary to DapuNPV (ML1 and T1 isolates) and OpMNPV. DijuNPV and HycuNPV do not possess this region. On the left—FastTree for the presented region alignment is shown

The region in between *trgf (tryptophan repeat gene family, ac30* homolog) and *fgf* (fibroblast growth factor) has been compared among five baculoviruses from genus Alphabaculovirus Ib—LesaNPV, DapuNPV, OpMNPV, DijuNPV, and HycuNPV (Fig. 6). LesaNPV genome lacks the fragment between genes *iap-3* and *ctl-2* (Fig. 6). This region in DapuNPV-ML1 and OpMNPV contains genes encoding small (*rrss*) and large (*rrls*) subunits of ribonucleotide reductase, *dUTPase* and small *orf* (*dapu129, op33*) of unidentified function. Another DapuNPV isolate—T1, from Turkey—is available in the GenBank database (accession number: OP891564). In general, it is almost identical to Polish isolate DapuNPV-ML1, only some single-nucleotide polymorphisms (SNPs) can be observed. They do not change the sizes and numbers of ORFs, except *rrss* sequence, which is shorter due to the deletion of two nucleotides in the middle of a sequence (Fig. 6). DijuNPV lacks the *rrss-dUTPase*, but the *sod* and *ctl-2* genes are similarly organized in its genome, like in LesaNPV, OpMNPV, and DapuNPV. However, *tmk* gene encoding for thymidylate kinase, which is involved in DNA synthesis occur in DijuNPV. This gene is in fusion with dUTPase in two mentioned baculoviruses (almost 75% identity in nucleotide sequences with the dUTPase fragment in *op31* and *dapu131*). HycuNPV lacks the region *rrss-dUTPase*, but *sod* and *ctl-2* have the opposite direction than in LesaNPV, DapuNPV, OpMNPV, and DijuNPV. HycuNPV, similarly to LesaNPV, lacks the region *rrss-dUTPase*. The phylogeny for this region (Fast Tree presented on the left in Fig. 6) is consistent with phylogeny of *Baculoviridae* family presented in Fig. 3.

**Fig. 7:**
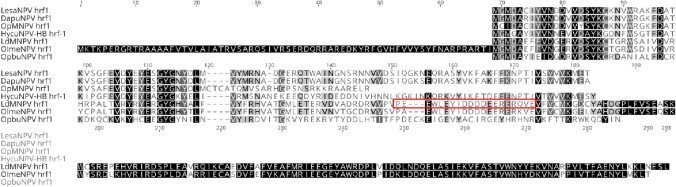
Host range factor-1 (HRF-1) amino acid sequences alignment from seven baculovirus isolates—LesaNPV, OpMNPV, DapuNPV, LdMNPV, OlmeNPV, ObpuNPV, and HycuNPV (China). Black indicates 100% similarity, a highly acidic region crucial for the function of HRF-1 (Ikeda et al., 2005) is marked with red rectangle

Functional *hrf-1 (host range factor-1*) from Lymantria dispar multiple nucleopolyhedrovirus (LdMNPV) is able to relieve translation arrest and promotes Autographa californica multiple nucleopolyhedrovirus (AcMNPV) replication in IPLB-Ld652Y cells (Ld652Y), a non-permissive L. dispar cell line [33]. This gene is present in LesaNPV genome and encodes for a protein of 109 aa long. Figure 7 presents the alignment of amino acid sequences of HRF-1 from baculovirus isolated from seven different hosts (representative sequences of baculovirus species were chosen). This protein sequence is almost identical (110aa, 96% of similarity) in LesaNPV and both isolates of DapuNPV (-ML1 and -T1), while is the shortest (78aa) in OpMNPV and lacks a C-terminal fragment. HycuNPV (Hyphantria cunea nuclepolyhedrovirus-HB and -BJ isolates, from China) HRF-1 have similar length to the protein sequence of LesaNPV and DapuNPV. HRF-1 was initially found in LdMNPV, homolog is also present in OpbuNPV (Operophtera brumata NPV; [34] and was recently discovered in OlmeNPV (Olene mendosa NPV [35]), which is closely related to LdMNPV. OlmeNPV HRF-1 is the longest, with 283aa in length and an additional fragment 68aa long on the N terminus, contrary to all the other six HRF-1 sequences. A highly acidic region of 20aa long, where 11aa are Asp(D) or Glu (E) residues, crucial for the function of HRF-1 was reported in LdMNPV sequence [33] (marked in red rectangle in Fig. 7). The same region is also present in OlmeNPV, but absent in LesaNPV HRF-1 sequence. Only one acidic residue Asp (D) at position 84aa occurs, conserved among all the analyzed HRF-1 sequences (except OpMNPV).

**Fig. 8:**
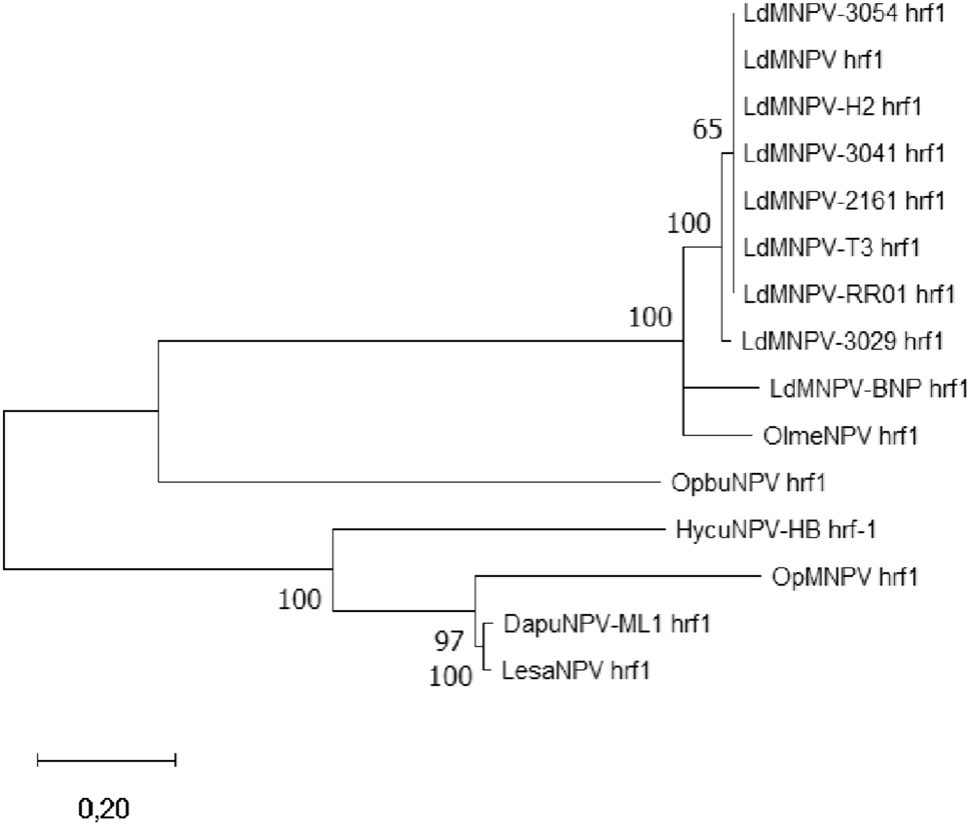
Maximum Likelihood molecular analysis of different amino acid sequences of HRF-1 available in GenBank database (isolates with 100% similar sequences were omitted in the phylogenetic tree). The percentage of 1000 bootstrap replicates is presented next to the branches

Maximum likelihood molecular phylogenetic analysis of host range factor-1 presented in Fig. 8 shows a pattern of clustering, where two clades can be distinguished consisting of LesaNPV, DapuNPV, OpMNPV, and HycuNPV-HB and the second—LdMNPV isolates, OlmeNPV, and ObpuNPV HRF-1.

## Discussion

Next-generation sequencing allows for deciphering newly discovered virus species or isolates genomes, which helps for better understanding the evolution of *Baculoviridae* family. The phylogenetic analysis suggests LesaNPV’s closer ancestral relationship compared to OpMNPV, underlining its potential broader host range. This hypothesis can be supported by the fact that in cross-reactivity assays, Douglas-fir tussock moth *Orgyia pseudotsugata* McDun. (Lepidoptera, Erebidae) larvae were infected by low doses of LesaNPV, while *L. salicis* larvae were not infected by OpMNPV even with 10^8^ polyhedra per larva. Additionally, *O. pseudotsugata* larvae were L3 instars, so they are not as susceptible to baculovirus infection as newly hatched L1 larvae (which were *L. salicis).* The explanation could be that LesaNPV has a broader host range than more specialized OpMNPV. Another puzzle is that only *L. salicis* is present in both areas, where *O. pseudotsugata* and *Dasychira pudibunda* L. (Lepidoptera, Erebidae) occur. The pale tussock moth (*D. pudibunda*) is present in the whole of Europe except in the Far North, but causes outbreaks only in the western and central part of the continent (between 48 and 57th parallels), while the Douglas-fir tussock moth can be found only in western North America [20, 36].

Previous analysis of fragments of LesaNPV genome using PCR technique [16] revealed the deletion of only dUTPase in the analyzed region, while gene encoding for ribonucleoside reductase large subunit (RR1) was detected. In our studies using whole-genome sequencing of LesaNPV, we have confirmed that it indeed lacks *dUTPase,* but in addition LesaNPV does not possess large and small RR subunits in the region between *iap-3* and *ctl-2* genes (Fig. 6). Ribonucleotide reductases (RRs) are built of two subunits large and small (rrls (RR1) and rrss (RR2)). They are responsible for converting ribonucleotides to deoxyribonucleotides (dNTPs) required for DNA replication and repair [37]. These ribonucleotide reductases have been found in granuloviruses, many group II alphabaculoviruses, and a few group I alphabaculoviruses (summarized by [38]). The two origins are possible for *rrls (rr1*) acquisition by baculoviruses: bacterium and eukaryotic cell (most likely insect cells) [39]. While, dUTPase function is preventing dUTP incorporation into DNA that could cause unwanted mutations [40, 41]. Homologs of *dUTPase* are present in many NPVs (most in Group II) and a few GV genomes. Baculoviruses may have acquired this gene to either supplement or substitute for the host gene [38]. Both proteins dUTPase and RR are responsible for the DNA repair system in baculoviruses. The *Baculoviridae* family members have independently acquired *dUTPase* and *rr* genes during their evolution [42]. Other closely related species HycuNPV and DijuNPV (however, possesses fragment of *op31/dapu131 dUTPase* (*tmk* gene) [43]) do not have the *rrss-dUTPase* region. It is considered that there is a selective advantage for viruses harboring the nucleotide metabolism genes [44]. Contrary to OpMNPV and DapuNPV, LesaNPV lacks certain genes, leading us to consider two potential scenarios: 1. an undiscovered baculovirus species may exist—an ancestor to all viruses discussed in this paper. 2. The LesaNPV isolate we studied either have lost this segment of its genome or OpMNPV and DapuNPV could gain this region, a phenomenon not uncommon within the entire family [42]. There has already been discussion that LesaNPV probably migrated together with the host *L. salicis* to North America and is an ancestor of OpMNPV or the second scenario where LesaNPV and OpMNPV have a common ancestor with a broader geographic distribution [16]. Further investigations using other isolates of the *Alphabaculovirus orpseudotsugatae* species could clarify this matter.

*Host range factor-1* was suggested to promote NPV infectivity (including SeMNPV, HycuNPV, BmNPV, and AcMNPV) of *Lymantria dispar* L. (Lepidoptera, Erebidae) cells and recombinant AcMNPV bearing-hrf-1 from LdMNPV exhibited increased infectivity toward *Helicoverpa zea* and *L. dispar* [45–47]. It is reported to be an essential factor in infecting *L. dispar* cells. Hrf-1 structure is important for its stability and function, highly acidic domain has been determined to play an essential role in the function of *hrf-1* gene [33]. Among over few hundred baculoviruses genomic nucleotide sequences available nowadays, only those isolated from hosts, like *L. dispar*, *Olene mendosa* Hübner, *Orgyia pseudotsugata*, *L. salicis*, *D. pudibunda* (Erebidae, Lymantriinae), *Hyphantria cunea* Drury (Erebidae, Arctiini), and *Operophtera brumata* L. (Geometridae) possess *hrf-1* in their genomes. This underscores the potential evolutionary significance of this factor in *Alphabaculovirus orpseudotsugatae* species or broader in Lymantriinae infecting baculoviruses and opens up new avenues for understanding the genomic complexity and diversity. The interesting fact is that *hrf-1* can be found in the genome of baculovirus isolated from Chinese *H. cunea* (HycuNPV-HB and -BJ; [48]), while baculovirus from the same host in Japan (HycuNPV) does not have this gene in its genome [49]. Both viruses isolated from the same host, but in different areas are classified as different baculovirus species [48].

Additionally, two lineages can be distinguished in the evolution of HRF-1 (Fig. 8)—“OpMNPV” and “LdMNPV,” as was previously reported [48]. It has been found that the highly acidic domain (79aa-98aa) present in LdMNPV HRF-1 is crucial for protein structure and function. OpMNPV HRF-1 is shorter, lacks this region, and does not allow for *L. dispar* cells infection by AcMNPV harboring *Ophrf1* gene [33]. Although LesaNPV HRF-1 (as well as DapuNPV and HycuNPV-HB) is longer than in OpMNPV, it does not possess this highly acidic domain.

Historically, baculoviruses has been named from the host that they were isolated from. Nucleotide sequence pairwise distances based on partial sequences of *polh/gran, lef8, and lef9* allows for affiliating newly discovered baculovirus isolates to proper species [8]. These highly conserved genes serves as a great tool for species demarcation among *Baculoviridae* family members. Recently, K2P values based on 38 core genes have been calculated and all the values for both—3 gene and 38 core genes—presented [32]. OpMNPV and DapuNPV were classified as isolates of a single species [32]. The 38 core gene K2P parameter, applied to each pair of analyzed viruses LesaNPV, OpMNPV, and DapuNPV showed a value lower than 0.021 (Table 1). This suggests that LesaNPV, OpMNPV, and DapuNPV should be classified as isolates of the same species found in different hosts, recently identified as *Alphabaculovirus orpseudotsugatae*, with historical origins in OpMNPV. However, phylogenetic studies considering all 38 core genes indicate that LesaNPV might be less divergent from a common ancestor than OpMNPV and DapuNPV. Taking into account the occurrence of these three viruses in the same area, a potential reclassification to *Alphabaculovirus lesalicis* should be considered. This finding could have significant implications for the classification and understanding of baculoviruses.

## Conclusion

Our results of whole-genome sequencing of LesaNPV confirmed that this baculovirus is a member of Alphabaculovirus group Ib and according to new nomenclature, should be assigned as *Alphabaculovirus orpseudotsugatae* species. The phylogenetic studies revealed that LesaNPV is less divergent from a common ancestor than OpMNPV and DapuNPV. LesaNPV, similarly to OpMNPV and DapuNPV, possesses *host range factor-1*, which can also be found only in a few other baculovirus species sequenced to date. We postulate that three analyzed baculoviruses are isolates of one species isolated from different hosts occurring in different areas.

### Supplementary Information

Below is the link to the electronic supplementary material.

Supplementary file1 (DOCX 92 KB)

Supplementary file2 (XLSX 19 KB)

## Data Availability

All data supporting the findings of this study are available within the paper and its Supplementary Materials. The complete LesaNPV genome characterized in this study is available under GeneBank accession number: OR759961.

## References

[1] Walker PJ, Siddell SG, Lefkowitz EJ, Mushegian AR, Adriaenssens EM, Alfenas-Zerbini P, Davison AJ, Dempsey DM, Dutilh BE, García ML, Harrach B, Harrison RL, Hendrickson RC, Junglen S, Knowles NJ, Krupovic M, Kuhn JH, Lambert AJ, Łobocka M, Nibert ML, Oksanen HM, Orton RJ, Robertson DL, Rubino L, Sabanadzovic S, Simmonds P, Smith DB, Suzuki N, Van Dooerslaer K, Vandamme A-M, Varsani A, Zerbini FM (2021). Changes to virus taxonomy and to the international code of virus classification and nomenclature ratified by the international committee on taxonomy of viruses (2021). Arch Virol.

[2] Blissard GW, Rohrmann GF (1990). Baculovirus diversity and molecular biology. Annu Rev Entomol.

[3] Blissard GW, Theilmann DA (2018). Baculovirus entry and egress from insect cells. Annu Rev Virol.

[4] Garavaglia MJ, Miele SAB, Iserte JA, Belaich MN, Ghiringhelli PD (2012). The ac53, ac78, ac101, and ac103 Genes are newly discovered core genes in the family baculoviridae. J Virol.

[5] Javed MA, Biswas S, Willis LG, Harris S, Pritchard C, van Oers MM, Donly BC, Erlandson MA, Hegedus DD, Theilmann DA (2017). Autographa californica multiple nucleopolyhedrovirus AC83 is a per os infectivity factor (PIF) protein required for occlusion-derived virus (ODV) and budded virus nucleocapsid assembly as well as assembly of the PIF complex in ODV envelopes. J Virol.

[6] Herniou EA, Olszewski JA, O’Reilly DR, Cory JS (2004). Ancient coevolution of baculoviruses and their insect hosts. J Virol.

[7] Jehle JA, Blissard GW, Bonning BC, Cory JS, Herniou EA, Rohrmann GF, Theilmann DA, Thiem SM, Vlak JM (2006). On the classification and nomenclature of baculoviruses: a proposal for revision. Arch Virol.

[8] Jehle JA, Lange M, Wang H, Hu Z, Wang Y, Hauschild R (2006). Molecular identification and phylogenetic analysis of baculoviruses from Lepidoptera. Virology.

[9] Rohrmann GF (1986). Polyhedrin structure. J Gen Virol.

[10] van Oers MM, Herniou EA, Jehle JA, Krell PJ, Abd-Alla AMM, Ribeiro BM, Theilmann DA, Hu Z, Harrison RL (2023). Developments in the classification and nomenclature of arthropod-infecting large DNA viruses that contain pif genes. Arch Virol.

[11] Haase S, Sciocco-Cap A, Romanowski V (2015). Baculovirus insecticides in Latin America: historical overview, current status and future perspectives. Viruses.

[12] Ziemnicka J (2008). Outbreaks and natural viral epizootics of the satin moth Leucoma salicis L. (Lepidoptera: Lymantriidae). J Plant Prot Res.

[13] Furniss RL (1939) Insects attacking forest products and shade trees in Washington and Oregon in 1937. Proc Ent Soc Brit Columbia 5–8

[14] Schaefer PW (1989) Diversity in form, function, behaviour, and ecology: an overview of the Lymantriidae (Lepidoptera) of the World. In: Proceedings „Lymantriidae: A Comparison of Features of New and Old World Tussock Moths” Eds. Wallner W.E. and McManus K.A. USDA, NEFS, Broomal, US, pp 1–19

[15] Wallner WE (1988) Diversity in form, function, behaviour, and ecology: an overview of the Lymantriidae (Lepidoptera) of the World. In: Proceedings „Lymantriidae: A Comparison of Features of New and Old World Tussock Moths” Eds. Wallner W.E. and McManus K.A. USDA, NEFS, Broomal, US, pp 65–80

[16] Jakubowska A, van Oers MM, Cory JS, Ziemnicka J, Vlak JM (2005). European Leucoma salicis NPV is closely related to North American Orgyia pseudotsugata MNPV. J Invertebr Pathol.

[17] Strokovskaya L, Ziemnicka J, Michalik J (1996). Genetic variability of four natural isolates of the Stilpnotia salicis multiple-enveloped nuclear polyhedrosis virus. Acta Biochim Pol.

[18] Ziemnicka J (1981). Studies on nuclear and cytoplasmic polyhedrosis viruses of the satin moth (Stilpnotia salicis L.) (Lepidoptera, Lymantriidae). Pr Nauk Inst Ochr Roślin.

[19] Ahrens CH, Russell RLQ, Funk CJ, Evans JT, Harwood SH, Rohrmann GF (1997). The sequence of theOrgyia pseudotsugataMultinucleocapsid nuclear polyhedrosis virus genome. Virology.

[20] Krejmer M, Skrzecz I, Wasag B, Szewczyk B, Rabalski L (2015). The genome of Dasychira pudibunda nucleopolyhedrovirus (DapuNPV) reveals novel genetic connection between baculoviruses infecting moths of the Lymantriidae family. BMC Genomics.

[21] Luft JH (1961). Improvements in epoxy resin embedding methods. J Biophys Biochem Cytol.

[22] Delcher AL, Bratke KA, Powers EC, Salzberg SL (2007). Identifying bacterial genes and endosymbiont DNA with Glimmer. Bioinforma Oxf Engl.

[23] Borodovsky M, McIninch J GeneMark: Parallel Gene Recognition for Both DNA Strands

[24] Rice P, Longden I, Bleasby A (2000). EMBOSS: the European molecular biology open software suite. Trends Genet TIG.

[25] Finn RD, Clements J, Eddy SR (2011). HMMER web server: interactive sequence similarity searching. Nucleic Acids Res.

[26] Minh BQ, Schmidt HA, Chernomor O, Schrempf D, Woodhams MD, von Haeseler A, Lanfear R (2020). IQ-TREE 2: new models and efficient methods for phylogenetic inference in the genomic era. Mol Biol Evol.

[27] Hoang DT, Chernomor O, von Haeseler A, Minh BQ, Vinh LS (2018). UFBoot2: improving the ultrafast bootstrap approximation. Mol Biol Evol.

[28] Jones DT, Taylor WR, Thornton JM (1992). The rapid generation of mutation data matrices from protein sequences. Comput Appl Biosci CABIOS.

[29] Kumar S, Stecher G, Li M, Knyaz C, Tamura K (2018). MEGA X: molecular evolutionary genetics analysis across computing platforms. Mol Biol Evol.

[30] Bideshi DK, Renault S, Stasiak K, Federici BA, Bigot Y (2003). Phylogenetic analysis and possible function of bro-like genes, a multigene family widespread among large double-stranded DNA viruses of invertebrates and bacteria. J Gen Virol.

[31] Kikhno I (2014). Identification of a Conserved Non-Protein-Coding Genomic Element that Plays an Essential Role in Alphabaculovirus Pathogenesis. PLoS ONE.

[32] Wennmann JT, Keilwagen J, Jehle JA (2018). Baculovirus Kimura two-parameter species demarcation criterion is confirmed by the distances of 38 core gene nucleotide sequences. J Gen Virol.

[33] Ikeda M, Reimbold EA, Thiem SM (2005). Functional analysis of the baculovirus host range gene, hrf-1. Virology.

[34] Harrison RL, Rowley DL, Mowery JD, Bauchan GR, Burand JP (2017). The Operophtera brumata Nucleopolyhedrovirus (OpbuNPV) Represents an Early. Divergent Lineage within Genus Alphabaculovirus Viruses.

[35] Harrison RL, Rowley DL (2022). The complete genome sequence of an alphabaculovirus from the brown tussock moth, Olene mendosa Hübner, expands our knowledge of lymantriine baculovirus diversity and evolution. Virus Genes.

[36] Goldmark P (2012) Douglas-fir tussock moth (Orgyia pseudotsugata): Outbreak status of a conifer defoliating caterpillar. USDA For Serv 1–6

[37] Reichard P (2002). Ribonucleotide reductases: the evolution of allosteric regulation. Arch Biochem Biophys.

[38] Rohrmann GF (2019) Selected baculovirus genes without orthologs in the AcMNPV genome: Conservation and function. In: Baculovirus Molecular Biology [Internet]. 4th edition. National Center for Biotechnology Information (US)

[39] Hughes AL, Friedman R (2003). Genome-wide survey for genes horizontally transferred from cellular organisms to baculoviruses. Mol Biol Evol.

[40] Baldo AM, McClure MA (1999). Evolution and horizontal transfer of dUTPase-Encoding genes in viruses and their hosts. J Virol.

[41] Vassylyev DG, Morikawa K (1996). Precluding uracil from DNA. Structure.

[42] Herniou EA, Olszewski JA, Cory JS, O’Reilly DR (2003). The genome sequence and evolution of baculoviruses. Annu Rev Entomol.

[43] Ribeiro BM, dos Santos ER, Trentin LB, da Silva LA, de Melo FL, Kitajima EW, Ardisson-Araújo DMP (2019). A nymphalid-infecting group I Alphabaculovirus isolated from the major passion fruit caterpillar pest Dione juno juno (Lepidoptera: Nymphalidae). Viruses.

[44] Ardisson-Araújo DMP, Lima RN, Melo FL, Clem RJ, Huang N, Báo SN, Sosa-Gómez DR, Ribeiro BM (2016). Genome sequence of Perigonia lusca single nucleopolyhedrovirus: insights into the evolution of a nucleotide metabolism enzyme in the family Baculoviridae. Sci Rep.

[45] Thiem SM, Du X, Quentin ME, Berner MM (1996). Identification of baculovirus gene that promotes Autographa californica nuclear polyhedrosis virus replication in a nonpermissive insect cell line. J Virol.

[46] Chen CJ, Quentin ME, Brennan LA, Kukel C, Thiem SM (1998). Lymantria dispar nucleopolyhedrovirus hrf-1 expands the larval host range of Autographa californica nucleopolyhedrovirus. J Virol.

[47] Ishikawa H, Ikeda M, Alves CAF, Thiem SM, Kobayashi M (2004). Host range factor 1 from Lymantria dispar Nucleopolyhedrovirus (NPV) is an essential viral factor required for productive infection of NPVs in IPLB-Ld652Y cells derived from L. dispar. J Virol.

[48] Peng X, Zhang W, Lei C, Min S, Hu J, Wang Q, Sun X (2022). Genomic analysis of two Chinese isolates of hyphantria cunea nucleopolyhedrovirus reveals a novel species of alphabaculovirus that infects hyphantria cunea drury (lepidoptera: arctiidae). BMC Genomics.

[49] Ikeda M, Shikata M, Shirata N, Chaeychomsri S, Kobayashi M (2006). Gene organization and complete sequence of the Hyphantria cunea nucleopolyhedrovirus genome. J Gen Virol.

