# Supplementary material for: Leucoma salicis nucleopolyhedrovirus (LesaNPV) genome sequence shed new light on the origin of the Alphabaculovirus orpseudotsugatae species
Source: Virus Genes. 2024 Apr 9;60(3):275–86. doi: 10.1007/s11262-024-02062-x (PMC11139710; doi:10.1007/s11262-024-02062-x)
Supplement: Supplementary file 1 — Supplementary file1 (DOCX 92 KB) [file 11262_2024_2062_MOESM1_ESM.docx]

***Leucoma salicis* nucleopolyhedrovirus (LesaNPV) genome sequence shed new light on the origin of the *Alphabaculovirus orpseudotsugatae* species**

Martyna Krejmer-Rabalska^1*^ (0000-0002-5263-6081), Lukasz Rabalski^1,2^ (0000-0002-5292-9263), Maciej Kosinski^1^ (0000-0002-5475-7733), Iwona Skrzecz^3^ (0000-0001-8542-2779), Jadwiga Ziemnicka^4^, Boguslaw Szewczyk^1^ (0000-0003-4957-4059)

^1^ Laboratory of Recombinant Vaccines, Intercollegiate Faculty of Biotechnology University of Gdansk and

Medical University of Gdansk, 80-307 Gdansk, Poland

^2^ Biological Threats Identification and Countermeasure Center, General Karol Kaczkowski Military Institute of Hygiene and Epidemiology, 24-100 Pulawy, Poland

^3^Department of Forest Protection, Forest Research Institute, 05-090 Sekocin Stary, Poland.

^4^Department of Biological Control and Quarantine, Institute of Plant Protection, 60-318 Poznan, Poland

*****Correspondence: [martyna.krejmer-rabalska@ug.edu.pl](mailto:martyna.krejmer-rabalska@ug.edu.pl)

**Supplementary material**


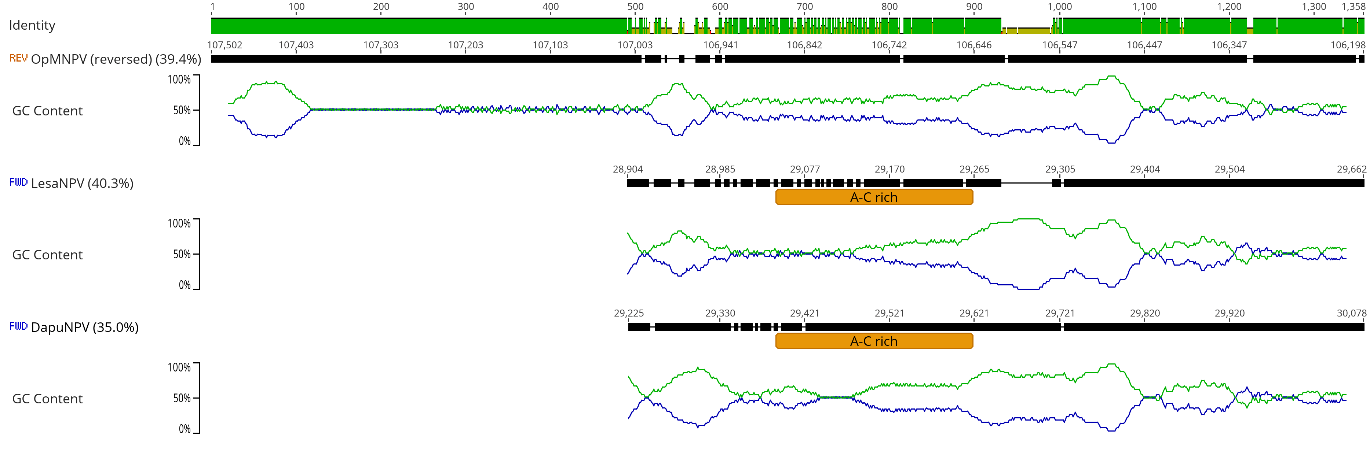


**Fig. S1.** An alignment of nucleotide sequences of the intergenic region between *p24* and *gp64* genes in OpMNPV (in reverse orientation), LesaNPV and DapuNPV. The identified AC-rich regions are annotated in orange. GC content in general in this fragment is presented near the sequence name on the right. The GC content is presented as green graph vs. AT content as blue graph. The green histogram represents consensus sequence identity. The numbers represent a position in each genome and the sequence length.
